# Molecular characterization and expression patterns of *Nanog* gene validating its involvement in the embryonic development and maintenance of spermatogonial stem cells of farmed carp, *Labeo rohita*

**DOI:** 10.1186/s40104-018-0260-2

**Published:** 2018-06-11

**Authors:** Swagat K. Patra, Chakrpani Vemulawada, Meenati M. Soren, Jitendra K. Sundaray, Manoj K. Panda, Hirak K. Barman

**Affiliations:** 1 Fish Genetics and Biotechnology Division, ICAR - Central Institute of Freshwater Aquaculture, Kausalyaganga, Bhubaneswar, Odisha 751002 India; 2 Center of Biotechnology, Siksha ‘O’ Anusandhan University, Bhubaneswar, India

**Keywords:** Embryonic stages, Gene expression, Nanog, Promoter analysis, Rohu (*Labeo rohita*), Spermatogonial cell

## Abstract

**Background:**

The homeobox containing transcription factor *Nanog* plays crucial roles in embryonic development/proliferation and/or maintenance of spermatogonial stem cells (SSCs) via interacting with transcription factors such as *Oct4* and *Sox2* in mammals. However, knowledge of its exact mechanistic pathways remains unexploited. Very little is known about teleost *Nanog*. Information on the *Nanog* gene of farmed rohu carp (*Labeo rohita*) is lacking. We cloned and characterized the *Nanog* gene of rohu carp to understand the expression pattern in early developmental stages and also deduced the genomic organization including promoter elements.

**Results:**

Rohu *Nanog* (*LrNanog*) cDNA comprised an open reading frame of 1,161 nucleotides bearing a structural homeodomain; whereas, the genomic structure contained four exons and three introns suggesting that it is homologous to mammalian counterparts. Phylogenetically, it was closely related to freshwater counterparts. Protein sequence (386 AA of 42.65 kDa) comparison revealed its low similarity with other vertebrate counterparts except that of the conserved homeodomain. Tissue distribution analysis revealed the existence of *LrNanog* transcripts only in adult gonads. The heightened abundances in the ovary and proliferating spermatogonia suggested its participations in maternal inheritance and male germ cell development. The potentiating abundances from fertilized egg onwards peaking at blastula stage vis- à-vis decreasing levels from gastrula stage onwards demonstrated its role in embryonic stem cell development. We also provided evidence of its presence in SSCs by western blotting analysis. Further, the promoter region was characterized, predicting a basal core promoter and other consensus elements.

**Conclusion:**

The molecular characterization of *LrNanog* and its documented expression profiling at transcript and protein levels are indicative of its functional linkage with embryonic/spermatogonial stem cell maintenance. This is the first report of *LrNanog* genomic organization including its promoter sequence information with predicted regulatory elements of a large-bodied carp species. This will be useful for elucidating its mechanism expression in future. Nanog could be used as a potential biomarker for proliferating carp SSCs.

**Electronic supplementary material:**

The online version of this article (10.1186/s40104-018-0260-2) contains supplementary material, which is available to authorized users.

## Background

Homeobox genes contain a characteristic Helix-Turn-Helix DNA-binding homeodomain encoding a motif of 60 or 63 amino acid (AA) residues [1]. The homeodomain is reported to be highly conserved across species featuring three consensus α-helices acting as a binding platform for DNA and an extended non-consensus N-terminal arm provides the basis of functional diversity [2]. Homeodomain-containing proteins are transcription factors (TFs) that regulate diverse developmental programmess by modulating expression patterns of targeted genes in a temporal, spacial and tissue-specific manner [3]. They are involved in cell identity/proliferation and also play a fundamental role in metazoan development [4].

*Nanog*, belonging to a member of the homeobox family, is believed to be a transcriptional activator. It binds to a 5′-TAAT-3′ core DNA motif. In mammals, it is believed to be associated with-finely tuned mechanistic pathways for maintenance of pluripotency and self-renewal of undifferentiated embryonic stem cells (ESCs) [5], even in the absence of leukaemia inhibitory factor (LIF) [6, 7]. *Nanog* specifically expressed in pluripotent cells of the mouse preimplantation embryo, embryonic germ cells and ESCs of murine [6–8] and human origin [4]. The mammalian TFs *Nanog*, *Oct4* (known as *Pou5f1* and *Pou2* in teleosts), and *Sox2* (Sex determining region Y box 2) constitute a complex reciprocal regulatory network (NOS network) that co-operatively maintains the self-renewal and stemness of ESCs [9–11]. *Nanog* should be a targeted factor for *Sox2-Oct4* synergism in pluripotent cells. It seems the expression of *Nanog* is regulated by *Oct4/Sox2* heterodimers, in which *Oct4/Sox2* binds to the octamer/sox elements within the *Nanog* proximal promoter region and induces *Nanog* transcription [5, 12, 13]. The exact mechanisms by which *Nanog* is specifically recruited to its binding sites, the mode of distinction between up-regulated and down-regulated targets; and the way *Nanog* signals to the RNA polymerase to either initiate or repress transcription are currently unresolved.

Since the last decade, extensive studies on ESCs have revealed/identified several basal TFs that aid the framework for retaining the pluripotency and stemness of these cells. As stated above, *Nanog* is considered one of the major TFs of the core pluripotency transcriptional network in mammals [5]. During mammalian embryonic development, it has been proposed to act as a selector gene during epiblast/primitive endoderm lineage decision [7]. Its overexpression confers LIF-independent self-renewal in mouse ESCs [6]. It was formerly proposed as a transcription repressor to inhibit the expression of genes important for cell differentiation. The lack of sufficient genotypic signatures and phenotypic features have been the bottleneck of Spermatogonial stem cells (SSCs)research advancement, specifically linked to the self-renewal and differentiating developmental stages in teleosts. Current progressions on purification and cultivation of teleost SSCs [14–17] provided a platform to undertake studies linked to the mechanistic networking pathways of self-renewal or differentiating features of male germ cell development. Recently, numerous studies were carried out on phenotypic features and/or genotypic signatures, such as *Thy1* (thymocyte differentiation antigen 1), *Pou2* (POU domain, class 2), *Plzf* (promyelocytic leukaemia zinc finger), *Gfr1*α (GDNF family receptor alpha 1) and *Ssea*-1 (stage-specific embryonic antigen 1), preferentially expressed in testes and proliferating undifferentiated spermatogonial cells of medaka (*Oryzias latipes*) [18], dogfish (*Scyliorhinus canicula* L.) [19] and rohu carp (*Labeo rohita*, a commercially important farmed carp) [14, 20, 21]. Interestingly, the existence and potentiating activities of *Pou2* and *Sox2* expressions were documented in long-term cultivated rohu spermatogonial cells [21, 22]. In teleosts, *Nanog* is reported to be a maternally inherited pluripotent gene and its documented gonadal presence during early embryonic development provides clues regarding its participation in regulation of proliferation of the developing embryos [23–26]. In zebrafish (*Danio rerio*), *Nanog* regulates the blastomeric division and germ layer patterning and thereby is proposed to be crucial for survival of early embryos [27]. In comparison with mammalian counterpart, functional dissection for teleost *Nanog* remained insufficient. Fishes, being one of the aquatic diversified species, it is essential to identify and characterize the species-specific *Nanog* gene, especially for commercially valuable farmed fishes. Even though enriched SSCs of farmed rohu carp (*L. rohita*) are capable of proliferating in vitro [14], information on the *Nanog* gene is lacking in this carp species. This prompted us to identify and characterize the *Nanog* gene of rohu carp (hereafter termed as *LrNanog*) so as to lay a foundation for advanced investigations.

In our present study, we identified and deduced the genomic organization including possible promoter elements of the *LrNanog*. Its relative expression patterns (mRNA) in the early embryonic developmental stages and various tissues including enriched SSCs (mRNA and LrNanog protein) are also documented.

## Methods

### Fish and embryos collections

This study was approved by the ethical committee of the ICAR-Central Institute of Freshwater Aquaculture, Bhubaneswar, Orissa, India. Adult rohu (*L. rohita*), (about 1-2 years old of weighing approximately 1 kg), were collected from the carp hatcheries of ICAR-Central Institute of Freshwater Aquaculture. The tissues, including the gonads (testis and ovary), kidney, spleen, heart, liver, intestine, brain, gill, skin and muscle were dissected aseptically from healthy adult rohu anaesthetized with MS-222 (100 μg/mL tricaine methane sulfonate, Sigma Aldrich, USA) [28]. Fertilized eggs were obtained by induced artificial fertilization and maintained at natural condition (pond water) with aeration. The embryonic developmental stages were observed under a microscope. Eight different embryonic stages (unfertilized eggs, 1 cell, 2 cells, 4 cells, 8 cells, 16 cells, 32 cells, blastula and tail-bud), hatching stage and 24 h of post-hatching stages) were selected. The embryos were microscopically dissected to remove the envelopes. The samples were frozen immediately in liquid nitrogen followed by storage at − 80 °C until processed for total RNA isolation.

### Cell culture

Rohu spermatogonial cells of undifferentiated nature were purified according to our previously established protocol [14, 22]. Enriched cells were cultivated in vitro for about 1 year in 0.2% (*wt*/*vol*) gelatinized flasks containing L15 (Invitrogen, USA) media supplemented with 10 mmol/L HEPES, 1 mmol/L sodium pyruvate, 1× minimum essential medium nonessential AA solution, 100 μmol/L 2-mercaptoethanol, 2 nmol/L sodium selenite, 6 mg/mmol/L *D*-(+)-glucose, 25 μg/mL insulin, 100 μg/mL transferrin, 0.5% BSA (fraction V), 10% foetal bovine serum, 100 μmol/L ascorbic acid (Sigma, USA), 10 ng/mL platelet-derived endothelial cell growth factor (Sigma, USA) and other supplements except Glial cell-derived neurotrophic factor at 28 °C in a humidified carbon dioxide (5%) incubator as described [14, 21, 22].

### Total RNA and genomic DNA isolation and cDNA synthesis

Total RNA was extracted from the sampled tissues and embryos using TRIzol™ RNA extraction reagent (Invitrogen, UK) following the manufacturer’s guidelines and described elsewhere [14, 21, 22, 29]. Total RNA was also extracted from the enriched rohu SSCs (up to 3 × 10^6^ cells) as above. RNA samples were treated with RNase-free DNase I (Invitrogen, UK) to eliminate the possibilities of DNA contaminations, purified, precipitated and quantified using the standard protocol. Extracted RNAs were verified by PCR using *β*-*actin* house-keeping gene primers. Genomic DNA was isolated from the liver of rohu via the phenol-chloroform extraction method [30–32]. The quantity and quality of extracted total RNA and genomic DNA were determined by agarose gel electrophoresis and Nanodrop readings. Total RNA (approximately 1 μg) was reverse transcribed using iScript cDNA Synthesis Kit (Bio-Rad, CA, USA) and Oligo (dT)_16_ as per the manufacturer’s guidelines.

### Cloning and sequencing of *LrNanog* mRNA

cDNA synthesized from testis mRNA was subjected to PCR using the primers (LrNG.F/R) listed in Additional file 1: Table S1, designed from the consensus sequence of *D. rerio*, *Carassius auratus*, *O. latipes* and other related species available in public databases (http://www.ncbi.nlm.nih.gov/) to obtain the partial sequence of *LrNanog*. The amplified fragment was isolated and purified using a Gel Extraction Kit (Qiagen, USA) and cloned into a pGEM-T easy vector (Promega, USA) and transformed into chemically competent *E. coli* DH5α cells. Sanger sequencing reactions were performed on the cloned-fragments using an automated ABI 3730 XL analyser. The sequence was verified as the partial cDNA sequence of *Nanog* using the BLASTn programme (https://blast.ncbi.nlm.nih.gov/Blast.cgi?PROGRAM=blastn&PAGE_TYPE=BlastSearch&LINK_LOC=blasthome) and aligned using the Clustal Omega (https://www.ebi.ac.uk/Tools/msa/clustalo/) program. The 5′- and 3′-ends were amplified using a SMARTer™ RACE cDNA Amplification Kit (Clontech, USA) following the manufacturer’s protocol and as described [21, 22, 28] using a gene specific primer (GSP) set (including nested GSP) as listed in Additional file 1: Table S1. The amplified fragments were processed for bidirectional sequencing as above. The deduced AA sequence generated by the ExPASy translate tool (http://expasy.org/tools/dna.html) was verified using the BLASTp program of NCBI.

### Sequencing of *LrNanog* promoter and genomic structure

The primers used for *LrNanog* genomic structure and the 5′-flanking region (upstream of the putative transcription start site (TSS) region) were designed from the generated mRNA sequence information. These genomic DNAs were amplified using a Genome Walker™ Universal Kit (Clontech, USA) with the help of the gene-specific primers (GSPs) designed from the known cDNA/genomic DNA sequence of *Nanog* sequence as per the manufacturer’s instructions and described earlier [21, 22]. All the gene specific primers are listed in Additional file 1: Table S1. The PCR products, extracted by a Gel Extraction Kit (Qiagen, USA), were cloned in pGEM-T easy vector (Promega, USA) and sequenced. The exon-intron structure of *LrNanog* was determined by aligning the obtained mRNA sequence with the genomic sequence. Bioinformatic analysis of promoter sequence and potential TF binding sites within the 5′ regulatory region of the *LrNanog* gene was mainly performed using the online program MatInspector (http://www.genomatix.de/matinspector.html) of Genomatix software suite version 3.5 and TRANSFAC (http://genexplain.com/transfac/). The potential transcription start site (TSS, + 1) was predicted by the Neural Network Promoter Prediction program (NNPP, http://www.fruitfly.org/seq_tools/promoter.html).

### Expression profiling of *LrNanog* mRNA by quantitative real-time PCR

The differential expression profiling of *LrNanog* gene in different tissues including SSCs as well as in different embryonic stages was performed in triplicate for each cDNA sample (three independent experiments) by quantitative real-time PCR (qPCR) using SYBR Green Real-time Master Mix II (Roche Diagnostics, Germany) in a Light Cycler® 480 II RT-PCR instrument (Roche Diagnostics, Germany) as per the manufacturer’s instructions and as described elsewhere [21, 22, 28]. Negative control reactions with respective RNA templates were performed to ensure efficient decontamination of genomic DNA. Because of the specificity and instability of gene expression during early embryonic development, we selected the two most stable reference genes (*β*-*actin* and *Elf1α*), based on our previous studies [20, 22], as internal controls so as to obtain more precise results. The *LrNanog* transcript-specific primers and the house-keeping gene primer sets were listed in Additional file 1: Table S1. The specificity of the primers was confirmed using the melting curve analysis followed by a high-resolution agarose gel electrophoresis to authenticate the presence of transcripts of exact sizes, and those were further confirmed by sequencing from both ends.

All the data of triplicate experiments were expressed relative to *β-actin,* which was used to normalize any difference in reverse transcriptase efficiency. Threshold cycle (C_t_) value (the PCR cycle number at which fluorescence was detected above the threshold and decreased linearly with increasing input target quantity) was obtained from the qPCR system software (Roche Diagnostics, Germany) and used to calculate fold change for the relative gene expression, using the Pfaffl method [33]. The significance of expression of the target gene was analysed using one-way ANOVA test in Microsoft Excel followed by a Student’s paired *t*-test. *P* < 0.05 was considered as statistically significant. All the data were expressed as means ±S.E.

### Western blotting

Tissue and spermatogonial cell extracts were prepared by lysing in buffer containing 1% Triton X-100, 140 mmol/L NaCl, 10 mmol/L Tris (pH 8.0) and protease inhibitor cocktail, followed by brief sonication [21, 22]. Western blot was performed using Nanog primary antibody (Cat. No. ab80892; Abcam, UK) following the protocoldescribed elsewhere with minor modifications [22, 34–36]. Briefly, the protein samples were suspended in SDS sample buffer (final concentration to 60 mmol/L Tris, pH 6.8, 2% SDS, 100 mmol/L dithiothreitol, and 10% glycerol) and boiled for 5 min. The protein samples were subjected to 10% SDS-PAGE, electrotransferred onto poly-(vinylidene difluoride) membrane (Millipore, India), and blocked with 5% skim milk in PBS before incubation with the primary antibody (1:500 dilution in PBS containing 5% skim milk). Antibody binding was detected using the respective secondary antibody conjugated with horse-radish peroxidase (1:2,000 dilution in PBS containing 5% skim milk) (Millipore, India) followed by 3,3′-Diaminobenzidine (DAB) staining (Sigma-Aldrich, USA). DAB staining was performed using SIGMA FAST 3,3′-Diaminobenzidine tablets (Sigma-Aldrich, USA) containing 0.7 mg/mL of DAB, 0.17 mg/mL of urea hydrogen peroxide and 0.06 mol/L Tris buffer in a dark box till bands were visible.

### In silico analysis of the rohu Nanog protein and 3D modelling

The deduced AA sequences of known Nanog proteins were retrieved from public databases. The evolutionary relationship of the *Nanog* gene of different species was analysed using the MEGA package version 6 software [37]. The accession numbers or Ensembl IDs of the protein sequences used in phylogenetic analyses are mentioned in the corresponding figure. The phylogenetic tree was built using Poisson Correction distance based upon the neighbour-joining method with 1,000 bootstrap replicates. The percent of similarity and identity of LrNanog protein with counterparts of other species were calculated using the MatGAT (Matrix Global Alignment Tool) Program [38]. Alignments of deduced AA sequence were achieved with the ClustalW Multiple Alignment program of BioEdit v7 [39]. The domain structure analysis was performed using the SMART program (http://smart.embl-heidelberg.de/). The secondary structure of LrNanog was predicted by SOPMA and the confidence level was checked in PsiPred (http://bioinf.cs.ucl.ac.uk/psipred/). Physioco-chemical data were generated from the ProtParam (http://web.expasy.org/protparam/) program of the ExPASy server.

In the absence of a suitable 3D structure in the PDB database, we have generated the tertiary structure of this Nanog protein by using the ab-initio modelling using PHYRE2 (http://www.sbg.bio.ic.ac.uk/phyre2/). In this server, we submitted a query sequence (FASTA) of Nanog protein for obtaining a 3D model and visualized by the PyMOL tool (http://www.pymol.org/). The structural quality assessment of predicted model of domain part of Nanog was carried out using the SAVEs tools (Structural Analysis and Verification Server; https://services.mbi.ucla.edu/SAVES/) [40, 41]. The refinement and overall quality of the model were performed using the ProSA (Protein Structure Analysis) web server (https://prosa.services.came.sbg.ac.at/prosa.php).

### Protein-protein interaction study and identifying post-translation modification sites present on Nanog protein

Interacting pathways of the Nanog protein with other proteins were depicted using a STRING database (https://string-db.org/cgi/input.pl). The scores of neighborhood, gene fusion, co-occurrence, co-expression and homology scores with the interacting proteins were considered.

Glycation sites of ε amino groups of lysine residues were predicted using a NetGlycate 1.0 server (http://www.cbs.dtu.dk/services/NetGlycate/). In NetGlycate, a score of N0.5 was considered as glycated. Phosphorylation sites were predicted using a NetPhos2.0 server (http://www.cbs.dtu.dk/services/NetPhos-2.0/). Serine, threonine, and tyrosine residues with a score of ≥0.5 were considered as likely phosphorylated AA. Ubiquitylation sites were predicted using a UbPerd (www.ubpred.org). Lysine residues with a score of ≥0.62 were predicted as ubiquitylated. Sumoylation sites were predicted using a SUMOplot (http://www.abgent.com/sumoplot). High probability motifs having a score of 0.5 were adjudged as possible sum oylated [42].

## Results

### Cloning and characterization of *LrNanog* cDNA and deduced protein sequence analysis

**Fig. 1:**
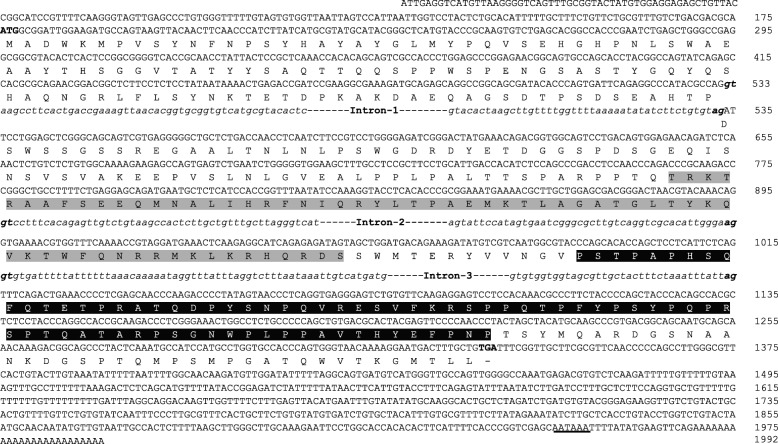
Nucleotide sequence of sequence structural organization of *Nanog* of *Labeo rohita*. The homeobox domain is shaded in grey background and the Proline-rich region in black background. The start and stop codons are in boldface. The intronic sequence are represented in lower case and italic font. The deduced amino acid sequences are shown underneath the CDS using single letter codes. The gt/ag as intron/exon boundaries are represented in bold and italic. Non-consensus polyadenylation signal (AATAAA) is underlined

In the absence of sequence information, *LrNanog* cDNA was partly (consisting of 712 nucleotides) cloned and sequenced from testicular and ovarian cDNA templates, using degenerate primers (Additional file 1: Table S1). The blast analysis showed maximum similarity/identity with the *D. rerio* and *C. auratus* counterparts (data not shown). Subsequently, the sequence information for the unknown terminal cDNA regions was generated by implementing 5′- and 3´-RACE strategies using gene-specific reverse and forward primers, respectively, designed from the above generated partial 712 bp sequence. The entire cloning strategy has been depicted in Additional file 2: Figure S1. Identifying overlapping RACE sequences, we were able to deduce a full-length cDNA of *LrNanog* is of 1992 nucleotides that comprised an open reading frame (ORF) of 1161 nucleotides along with both 5′-untranslated region (UTR) of 175 nucleotides and 3´-UTR of 656 nucleotides. The 3´-UTR contained a putative polyadenylation signal (AATAAA) 17 nucleotides upstream of the poly (A) tail (GenBank Accession Number: KX268304) (Fig. 1). The ORF initiated with an ATG start codon fulfilling the consensus Kozak criterion (A/GNNATGG) of eukaryotic translation initiation [43], while terminated with a TGA stop codon. The putative translated 386 AA of *LrNanog* has a predicted molecular weight of 42.65 kDa with an estimated isoelectric point of 7.17. In line with earlier evidence, the *LrNanog* ORF contained a characteristic 63 AA long homeobox (HOX) domain positioning between 197 and 259 AA [26] including a proline-rich motif spanning 272-347 AA (Fig. 1). These above findings revealed the existence of expressed *Nanog* gene in the farmed rohu carp, *L. rohita*.

**Fig. 2:**
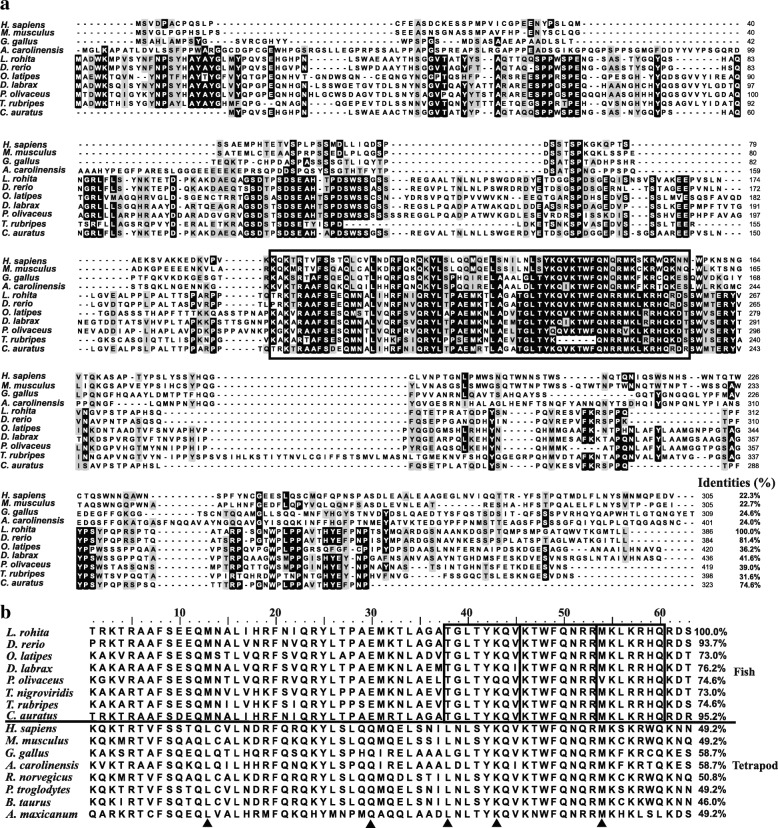
Multiple alignment of the LrNanog protein. **a** Multiple alignment of full-length LrNanog deduced amino acid sequences with other species counterparts. The conserved homeodomain sequence is denoted within a box. **b** The homeodomain amino acid sequence of LrNanog with other counterparts. Identity scores in respect to rohu homeodomain are shown on the right. The alignment was generated by using BioEdit ClustalW program

Multiple sequence alignments for the deduced full-length Nanog proteins of available tetrapod and teleosts revealed that LrNanog shared a relatively higher sequence identity with *D. rerio* (accession no.NP_001091862) and *C. auratus* (accession no. AEG74407) in the tune of 81.4 and 74.6% respectively, with considerably lower identity with human (accession no. NP_079141) counterpart (Fig. 2a). It is noticeable that the N- and C- termini of Nanog proteins were highly variable except that of the conserved homeodomain (HOX) motif. In addition, the HOX of LrNanog exhibited identities ranging from 46 to 95.2% to the counterparts of other vertebrate proteins (Table 1). Methionine (M13), glutamic acid (E30), and threonine (T38) residues were noticed to be conserved among fish species; respectively; in place of leucine (L13), glutamine (Q30) and leucine (L38) in tetrapods. In addition, three regions were identified as nearly conserved in teleosts (boxed in Fig. 2b). A few AA residues, such as those of methionine (M31), alanine (A35), glycine (G39), threonine (T41), arginine (R53), leucine (L57), histidine (H59) and aspartic acid (D62), were found to be conserved only in fish species. In addition, some AA residues, such as arginine (R5), glutamine (Q12 and Q23), phenylalanine (F20), leucine (L34 and L40) and lysine (K55). were found to be conserved in teleosts and tetrapods.

**Table 1:** Similarity (%) and identity (%) of rohu Nanog as well as its domains with other species

	Total amino acid		Homeodomain	
	Similarity	Identity	Similarity	Identity
*Danio rerio*	86.8	81.4	98.4	93.7
*Caracius auratus*	77.5	74.6	98.4	95.2
*Oryzias latipes*	52.6	36.2	85.7	73.0
*Dicentrarchus labrax*	56.9	41.6	88.9	76.2
*Paralichthys olivaceus*	54.7	39.0	85.7	74.6
*Tetradon nigroviridis*	49.0	30.2	84.1	73.0
*Takifugu rubripes*	52.0	31.6	84.1	74.6
*Homo sapiens*	33.9	22.3	68.3	49.2
*Mus musculus*	36.5	22.7	68.3	49.2
*Gallus gallus*	37.3	24.6	79.4	58.7
*Anolis carolinensis*	39.2	24.0	79.4	58.7
*Rattus norvegicus*	36.8	23.0	68.3	50.8
*Pan troglodytes*	33.9	22.5	68.3	49.2
*Bos taurus*	35.8	21.7	68.3	46.0
*Ambystoma mexicanum*	33.2	22.8	60.3	49.2

### Evolutionary relationship of Nanog among mammals and teleost

**Fig. 3:**
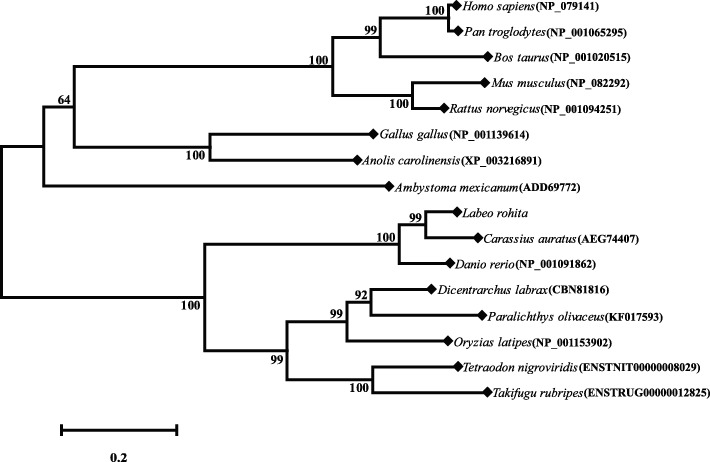
Phylogenetic analysis of LrNanog. The evolutionary relationship with other Nanog protein counterparts were verified by using MEGA 6.1 program by the bootstrap method of neighbor-joining with 1,000 replicates. The bootstrap values are mentioned next to the branches. Accession numbers of each protein sequence are specified in parenthesis

To evaluate the evolutionary relationships, a phylogenetic tree was constructed from the deduced AA sequence of full-length protein using the neighbour-joining method with 1,000 replicates (Fig. 3). The teleost and mammalian Nanog proteins are grouped into two distinct clades with above 60% bootstrap support. In the teleost clade, the fresh- and brackish-water species formed two separate clusters. Among the freshwater teleost cluster, LrNanog protein clustered with *C. auratus* and *D. rerio* with maximum bootstrap values of 99% and 100%, respectively (Fig. 3). The marine teleosts including *Tetraodon nigroviridis* and *Takifugu rubripes*, formed a separate cluster, whereas the brackish-water species, including *Dicentrarchus labrax*, *Paralichthys olivaceus* and *O. latipes*, formed a separate tight cluster. Even though *O. latipes* is known to be a freshwater species, its habitat also has been reported in salt water [44, 45]. In the mammalian clade, *Homo sapiens, Pan troglodytes* and *Bos taurus* showed a tight cluster, whereas two rodents such as *Mus musculus* and *Rattus norvegicus* separately clustered. The Nanog of *Gallus gallus*, *Anolis carolinensis* and *Ambystoma mexicanum* clustered in between mammals and teleosts, which was in line with the previous findings [26].

The comparative analyses for both deduced protein sequence and homeodomain of LrNanog exhibited high degree (> 70%) of similarity/identity with *C. auratus* and *D. rerio* counterparts (Table 1). The LrNanog protein showed merely ~ 50% similarity with the other teleosts, while least with mammalian counterparts. However, the homeodomain was found to be almost conserved among teleosts (> 80% sequence similarity) compared with other vertebrates. The homeodomain showed the highest levels of identity and similarity with *C. auratus* and *D. rerio* (≥ 93%).

### Genomic structure including possible promoter of *LrNanog*

**Fig. 4:**
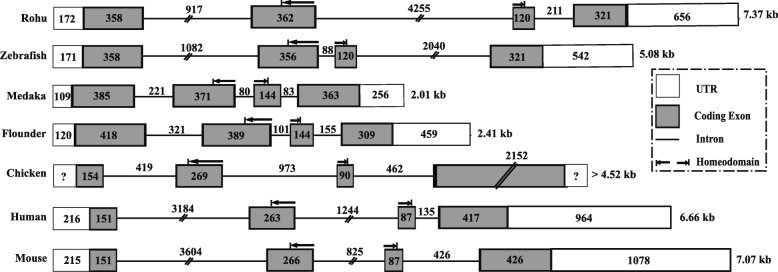
Comparison of genomic organization of *LrNanog* with other *Nanog* counterparts of teleost and mammals. Exons are shown in dark box whereas introns are in straight line. The size of each parts are indicated. The 5′- and 3′- Untranslated regions (UTR) are represented by open box. The 5′- and 3′- region of chicken are not available

Intron–exon boundaries were derived by aligning cDNA and genomic DNA sequences of LrNanog. It contained four exons and three introns (Fig. 4). All exon–intron boundaries conformed to a 5′-GT/3′-AG splicing rule. Similar to other teleostean and mammalian counterparts, *LrNanog* gene contained four exons. However, gene sizes in terms of both exons and introns varied from species to species. Its conserved homeodomain region was found to be located between the second and third exons. The above sequence information also detected the TSS (adenosine nucleotide) as marked in Fig. 1.

**Fig. 5:**
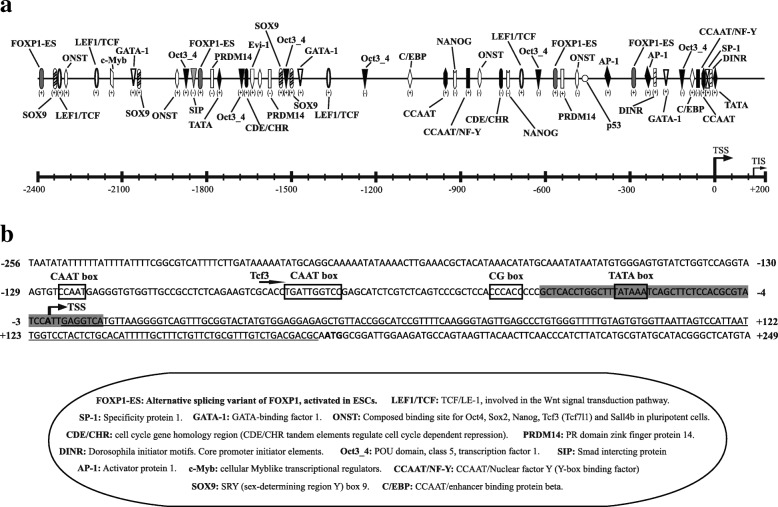
Schematic representation of *LrNanog* promoter region. **a** A diagrammatic presentation of predicted putative transcription factors/regulatory motifs of *LrNanog* gene. The scale is given and the plus-minus signs indicates the transcription factor (TF) binding strand. The Transcriptional start site (TSS; + 1) is represented by a tall arrow and the Transcription initiation site by a small arrow. The full name of the TFs are given at the bottom. **b** The predicted putative basal core promoter sequence as point out in (**a**). The promoter region predicted by NNPP is shadowed. The putative core promoter elements are boxed and indicated. The 5′-untranslated region is marked with a straight line. The TSS and start codon are highlighted in bold

The 2,462 bp upstream sequence, beyond the TSS, was analysed to predict the promoter region using computational tools. We could predict a putative basal core promoter containing numerous transcriptional elements, such as, a TATA-box located at − 22 to − 27 bp, a GC-rich box spanning − 44 to − 49 bp, and two CAAT-boxes located at − 79 to − 83 bp and − 120 to − 124 bp upstream of the TSS (Fig. 5a and b). The presence of several other *cis*-acting modules/platforms for various TFs such that of Nanog, SIP, PRDM14, FOXP1-ES, LEF1/TCF, SOX/SRY-sex/testis determining and related HMG box, CCAAT/enhancer binding protein (C/EBP), GATA binding protein (GATA-1), activator protein-1 (AP-1), specificity protein 1 (Sp-1), CCAAT/NF-Y and transcriptional activator c-Myb were also predicted (with high matrix weights ranging from 0.9 to 1.0) in the surrounding vicinity of the core promoter element (Fig. 5a). As it is known that Nanog is a TF responsible for maintaining the pluripotency, in this study numerous *cis*-elements important for pluripotency of ESC were identified with high matrix weights ranging from 0.9 to 1.0.

### *Nanog* is expressed prominently in rohu proliferating SSCs and is maternally inherited

**Fig. 6:**
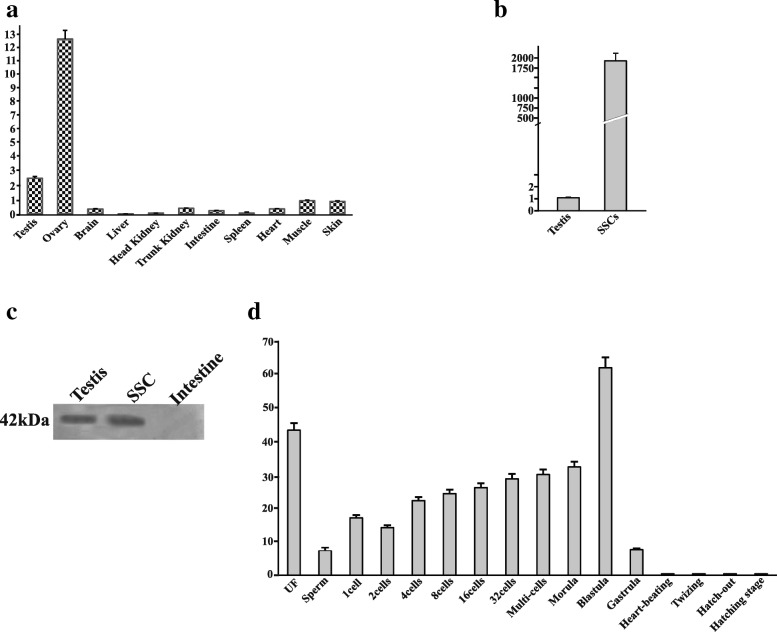
Expression profiling of *LrNanog* gene. **a** Relative expression profiling of *Nanog* in different tissues of *L. rohita* by quantitative real-time PCR. The data in each tissue was normalized with the value of the reference genes. **b** Relative expression profiling of *Nanog* in proliferative spermatogonial stem cells of rohu with comparison to testis. **c** Western blot showing the presence of Nanog in the testis and in vitro propagated spermatogonial stem cells of rohu with a signal at 42 kDa and intestine (un-related organ) was taken as a control. **d** *Nanog* expression during embryonic development from unfertilized eggs to hatching stage in *L. rohita*. All the data represent the mean of three independent experiments (each in triplicate). Data are shown as mean ± SE

The involvement of *Nanog* in male germ cell development in teleosts remained unclear. Hence, we examined the expression profile of *LrNanog* transcript in rohu (*L. rohita*) dividing SSCs including other organs using qPCR. *LrNanog* mRNA abundance was documented in the ovary and testis only with negligible detected levels in other somatic tissues including heart, brain, liver, kidney (both head and trunk), intestine, spleen, muscle and skin (Fig. 6a). Furthermore, the signal was relatively stronger (12.5 fold) in ovary than testis. Such an expression pattern was also observed in other species such as Japanese flounder, goldfish and medaka [23–26]. The *LrNanog* mRNA was most abundantly expressed in proliferating SSCs in tune of 1,978-fold more than testis (Fig. 6b). The presence of LrNanog protein was also documented in rohu testis and proliferating SSCs as detected by Western blot analysis with a specific signal of about 42 kDa (Fig. 6c), providing an insight into its involvement in stem cell maintenance and development.

The progressive expression pattern of *LrNanog* during early stages of embryonic development was also examined. We found that *Nanog* mRNA in rohu was detected in metaphase II oocytes (i.e. unfertilized eggs) (Fig. 6d) and at all stages preceding the embryonic genomic activation that occurs during blastula. Its elevated level suddenly decreased following the gastrulation stage, possibly because the pluripotent cells started differentiating [46]. These outcomes are in line with the previous data obtained in medaka, goldfish and Japanese flounder [23–26]. The *LrNanog* transcripts were impressively moderated at the end of the gastrulation stage. From the heart-beating stage onwards, *LrNanog* could not be documented. Together, our results postulated that the *Nanog* transcript is most likely inherited maternally and functionally linked to embryonic development, in addition to its participatory roles in SSC maintenance in teleost.

### Modelling and validating the structure of the homeobox domain of Nanog protein

The secondary structure predicted by SOPMA documented the absence of beta-sheets in LrNanog protein. It predicted 20.21% alpha helix, 62.18% random coils, 11.66% extended strands and 5.96% beta turns. The confidence level of the predicted secondary structure was determined using PsiPred (Additional file 3: Figure S2).

**Fig. 7:**
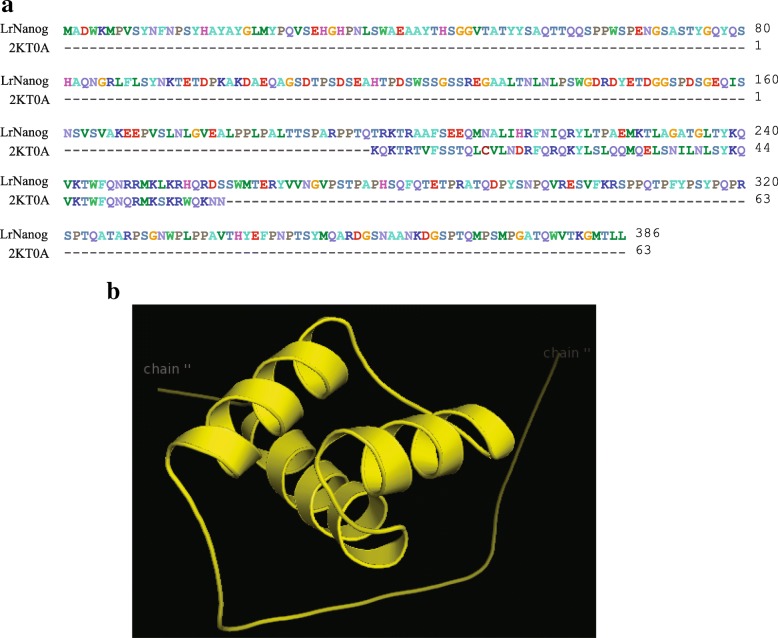
Predicted tertiary structure of the LrNanog protein. **a** Sequence alignment of LrNanog protein sequence with the template (2KT0A) of *H. sapiens*. **b** Based on the template sequence the protein model of LrNanog was generated by means of ab-initio modelling using PHYRE2

The 3D structure of teleost Nanog protein has not yet been determined experimentally. In the absence of crystal structure (3D structures in the PDB database), the in silico analysis is used for the prediction of protein structure to characterize a new protein. We attempted to build a protein structure by *abinitio* modelling using PHYRE2. The model building was based upon the template 2KT0A (human stem cell TF Nanog homeodomain) that covered a total of 63 residues positioning Thr197 to Lys259, out of a total of 386 AA residues of LrNanog protein (Fig. 7a). After rigorous refinements by means of the electron microscopy technique, a stable structure of the HOX domain could be built (Fig. 7b).

The quality of the predicted structured model was assessed/validated by Ramachandran plot in the PROCHECK validation package (Additional file 4: Figure S3). In the plot, the phi/psi angles for 96.7% residues were with the ‘most favoured’ regions inclusive of 3.3% residues in the ‘additional allowed’ regions, while none of the residues lay in the ‘disallowed conformations’. This indicated that the backbone dihedral angles, phi and psi, in the LrNanog HOX domain model were reasonably accurate. Further, the overall PROCHECK G-factor for the structure was 0.10, implying that the modelled structure is acceptable. The value of the Z-score signified that the 3D model of the HOX domain of LrNanog protein was reliable. Energetic architecture as revealed by ProSA (data not presented) score was negative (− 4.3) for the modelled protein. ProSA and Errat analysis implied that the Z-score of our model was very much within the range of scores normally found for proteins of comparable size. The results, together, ascertained the qualities of a stable and reasonably good model structure.

### Protein-protein interactions for Nanog and posttranslational modification on LrNanog protein

**Fig. 8:**
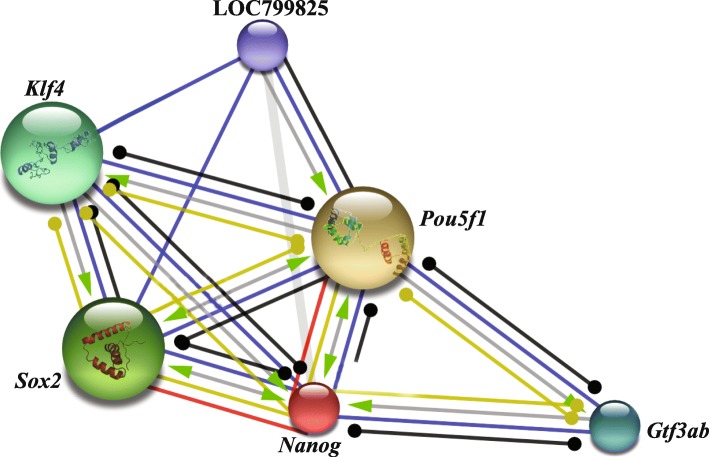
Interaction network between Nanog and other factors/proteins involved in the pluripotency, as predicted by STRING

*Nanog* is a main member of the NOS triad responsible forstem cell pluripotency and maintenance. Protein-protein interacting networks of Nanog along with other factors (Table 2; Fig. 8) involving pluripotency was investigated using STRING. This revealed that LrNanog protein interacted with the other pluripotency marker proteins such as, Pou5f1 (POU domain, class 5, TF1), Sox2, Klf4 (Kruppel-like factor 4), Gtf3ab (general TF IIIA, b) and LOC799825 (lin-28 homolog A) with scores of 0.996, 0.997, 0.937, 0.909 and 0.908 (Table 2), respectively, that provided a clue regarding its involvement in pluripotency and maintenance of stem cells.

**Table 2:**
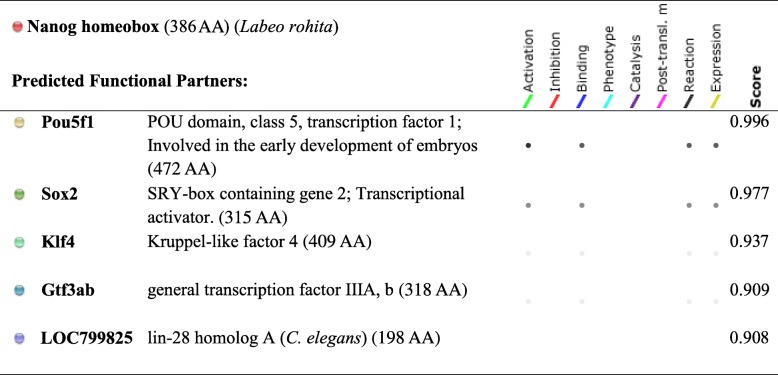
Protein-protein interacting networks as determined by STRING

We used different in silico tools to identify/study post-translational modifications of LrNanog protein. NetGlycate predicted that 7 AA residues would undergo glycation. As per NetPhos analysis 30 serine, 13 threonine and 8 tyrosine residues would undergo phosphorylation (Table 3). Similarly, UbPred, showing 5 AA positions, had a score of above 0.84, indicating a high possibility of ubiquitination, whereas 1 AA position showed a medium chance of ubiquitination. Likewise, by using SUMOplot, we were able to predict two positions where there was a moderate level of the chance of sumoylation (Table 4).

**Table 3:** Post-translation modified glycation and phosphorylation sites in LrNanog protein

Glycation		Phosphorylation									
N-Gly		Serine						Threonine		Tyrosine	
Pos	Score	Pos	Score	Pos	Score	Pos	Score	Pos	Score	Pos	Score
5	0.938	29	0.962	124	0.609	259	0.994	58	0.589	16	0.912
99	0.885	37	0.962	126	0.992	260	0.982	110	0.910	43	0.890
167	0.583	62	0. 756	127	0.965	279	0.985	118	0. 534	75	0.621
239	0.926	66	0.993	140	0.913	301	0.965	187	0.687	146	0.826
242	0.773	73	0.958	152	0.979	306	0.984	197	0.937	266	0.953
251	0.896	108	0.847	155	0.987	315	0.655	223	0.935	293	0.946
253	0.910	112	0.990	162	0.799	321	0.882	237	0.624	342	0.681
		114	0.938	164	0.788	330	0.940	263	0.740	350	0.669
		121	0.987	189	0.840	349	0.658	285	0.979		
		123	0.924	205	0.958	365	0.985	289	0.886		
								310	0.856		
								326	0.910		
								340	0.955

**Table 4:** Putative ubiquitylation and sumoylation sites in Nanog protein

Ubiquitylation			Sumoylation	
Residues	Score	Ubiquitinated	Residue	Score
93	0.87	High confidence	5	0.54
99	0.85	High confidence	169	0.79
101	0.89	High confidence		
167	0.90	High confidence		
228	0.63	Low confidence		
362	0.94	High confidence		

Score range: low confidence –0.62 ≤ s ≤ 0.69, medium confidence –0.69 ≤ s ≤ 0.84, high confidence 0.84 ≤ s ≤ 1.00

## Discussion

*Nanog* gene, in association with *Oct4* and *Sox2* genes, is believed to regulate the stemness properties of mammalian stem cells. Presently, its specific underlying mechanisms for pluripotency and maintenance of stem cells are not well understood in teleost fishes. *Nanog*, as a pluripotent gene, has been characterized in mammals [6] and non-mammalian species [47]. Its orthologs have been characterized in teleostean model organisms, such as zebrafish and medaka [23, 48], including aquarium goldfish [25]. The genomic information for the *Nanog* gene of large-bodied farmed rohu carp (*L. rohita*) was lacking. The current study focused on the identification and characterization of *Nanog* ortholog in rohu carp. We were able to elucidate the *Nanog* gene structure along with its promoter modules and tissue/cell distributions.

The full-length cDNA of *LrNanog* of 1,992 nucleotides comprised of a coding sequence of 1,161 nucleotides translatable to a polypeptide, of 386 AA residues. It contained a typical characteristic 63 AA containing HOX domain. The deduced AA sequence of LrNanog showing least homology to other mammalian species, even with other teleost species, demonstrated that *Nanog* is not highly conserved across mammalian and aquatic species under study. The protein comparison of LrNanog with other species counterparts demonstrated its sequence variations amonga wide range of organisms, except that of a relatively conserved HOX-homeodomain. Similar findings were also reported earlier [6, 23]. The HOX domain of *LrNanog* lacked the conserved tryptophan (W) pentapeptide repeats in the C-terminus region as documented in human and mouse [4, 6, 49]. The W-repeats were also not found in lower vertebrates of Japanese flounder, chick and zebrafish [26, 47, 48].

Phylogenetic analysis revealed that the LrNanog formed a distinct hierarchy along with the other teleost counterparts depending on habitats (fresh-, brackish- and marine-water), in which the LrNanog was found to be the most closely related to *C. auratus* and *D. rerio* counterparts. This is possible because *L. rohita*, *C. auratus* and *D. rerio* belong to a common family (Cyprinidae) of freshwater habitat. Tetrapods and teleosts formed different clusters, indicating a rapid evolution of the *Nanog* gene that might have begun before the separation of the teleost and tetrapod lineages. These results, together, suggested the W-repeats might have appeared in the higher mammals during evolution. The overall conserved HOX domain, except for W-repeats, possibly evolved from a common ancestor. It is also indicative that its stemness function is associated across species. The overall sequence variations could indicate that its participatory regulatory functions in stem cell maintenance may not be identical, but differ/vary from lower to higher vertebrates (species to species). The lack of W-repeats in conserved HOX vis-à-vis three identified conserved regions in teleosts as comparedwith mammalian counterparts provided the clue that the binding platform with other important regulatory molecules for teleostean *Nanog* is less likely to be exactly similar to that of the mammalian counterpart. In such a scenario, two distinct *Nanog*-mediated networking pathways must be operative between teleosts and mammals.

A total of 9.8 kb long genomic structure, encompassing four exons and three intronic sequences, was generated. Based on genome walking and 5´-RACE data, the transcription start site (TSS, + 1) with adenosine nucleotide (174 bp upstream from the start codon ATG) was identified. This was in line with verified in silico data. The genomic structure of *LrNanog* was found to be conserved in terms of the number of exons and the position of the homeodomain with other counterparts that promoted the probable degree of conservative function of the homeodomain. The larger coding exons in teleosts than mammalian counterparts demonstrated the possible rearrangement of *Nanog* gene during evolution from fish to tetrapod. The sizes and sequence differences of intronic regions among various species also reflected differential regulatory mechanisms because introns are known to participate in regulating gene expressions.

The in silico sequence analysis of the 5′–flanking genomic region led to the identification of consensus TATA- and CAAT-boxes along with GC-box, which is in line with the previous findings in goldfish and Japanese flounder [25, 26], but contrary to the previously documented absence in mouse *Nanog* [50]. *LrNanog* has a conventional RNA transcription polymerase II binding site (TATA-box) right upstream (22 bp) of the putative TSS. From these findings, it might be predicted that the 250 bp upstream from the TSS would be the core promoter region responsible for the *LrNanog* transcriptional activity. In addition, it is likely that the *LrNanog* gene is driven by a relatively compact regulatory region, because the full-length promoter of rohu (2.4 kb) is about half of the mammalian *Nanog* promoter (~ 5 kb) [50]. Unfortunately, we could not detect any mammalian type consensus sequence as a binding platform for the *Oct4/Sox2* complex [12], which is believed to be the major regulatory element. However, the identified *Oct4* binding motif provided the clue regarding its associated regulatory function with teleost *Pou2* ortholog. Interestingly, a p53 motif that down-regulates *Nanog* expression in ESCs during differentiation [51] and Tcf3 binding consensus sequence that limits its self-renewal function [52] were also detected in the *LrNanog* promoter. Its own binding site on the promoter region indicated that *Nanog* not only forms a homodimerization to promote stem cell pluripotency [53] but also could regulate its own expression. Furthermore, some other transcriptional activators (AP-1, SP1, GATA-1 and C/EBP) were also detected in the promoter region that could regulate the expression of *Nanog* via interacting with the other TFs and enhancers as suggested earlier [54]. It would be interesting to ascertain the regulatory functions played by each motif in the future. Even though the *Sox2* site could not be predicted, we could detect other SRY (sex-determining region Y)-box factors (Sox5, 6, 7, 8, 9, 21) on the promoter region indicating the potential role of *Nanog* in the regulation of embryonic and sexual development allied with its mRNA distribution profile. Furthermore, we were able to predict some TFs, including Oct3_4, SIP, PRDM14, FOXP1-ES, LEF1/TCF and ONST that are reported to be involved/associated with stem cell pluripotency [24, 55–57]. The computational analysis of TFs is mainly based upon the sequence similarity, so further experiments are necessary to draw any conclusion regarding the functional involvement of these TFs in the regulation of rohu *Nanog* gene expression.

The model structure of LrNanog HOX showed that the alpha helix covered 20.21% of total length and there are no beta-sheets. The value of the Z-score signified the 3D model of the Nanog protein was reliable and precise. The characteristic of good quality model protein was further evident from the Ramachandran plot. This validated that the predicted model protein was well inside the range of typical native structures. Furthermore, the protein-protein interactions, inclusive of pluripotency factors as previously reported in mammals [58], predicted the possible participation of LrNanog in the networking mechanisms pertaining to pluripotency stem cells. Several post-translational modifications were identified in the LrNanog protein.

Organ-wise gene expression analyses revealed that *LrNanog* gene was transcriptionally active only in male and female germ cells of the adult. In addition, the temporal mRNA expressions of *LrNanog* during early stages of embryonic development exist from fertilized xegg to blastula stage. *LrNanog* transcript expression showed lower levels during cleavage stages. Documented incremental abundances starting from unfertilized/fertilized eggs up to dramatically heightened blastula stage followed by a sudden decline in gastrula stage and subsequent absence up to hatchlings revealed its associated physiological function for undifferentiated ESCs. A similar stochastically increased expression pattern was also observed during mouse embryonic development [59]. This is possible because the blastula stage of teleosts comprises the maximum number of pluripotent cells and those differentiate into specific lineages at the beginning of gastrulation. Thus, it is likely that the participatory transcriptional regulation of *LrNanog* is associated with genome activation of ESCs. This is contrary to the fact that the primary function of Nanog is restricted to the proper formation of the extra-embryonic yolk syncytial layer [60]. The regulated fate of the *LrNanog* beyond blastula stage remains a mystery, which needs to be resolved in future. Similary to the ESCs, it is also involved in maintaining proliferating SSCs in testes as evident from this study. Its documented expression patterns (both transcript and protein levels) in proliferating SSCs demonstrated that it could also be a biomarker for rohu proliferating spermatogonial cells of undifferentiatednature. Our results also suggested that *LrNanog* actively participates in self-renewal of SSCs via finely tuned mechanistic pathways involving *Pou2* and *Sox2*, because *Pou2* and *Sox2* are also highly expressed in rohu SSCs [22, 61]. Thus, a mammalian-like networking function could not be ruled out. The potentiating presence of *LrNanog* mRNA in the ovary could be argued as its maternal inheritance, as also reported in other teleostean species [23–26, 48]. These observations also supported the computational protein–protein interaction findings. A population of enriched undifferentiated rohu SSCs also produces [14] spermatids in vitro vis-à-vis its abundance in oocytes, and thus the participatory role of *LrNanog* in undifferentiated germ cell development cannot be ruled out. It would be of interest to clarify this particular aspect in future. Because *Pou2* (an ortholog of mammalian *Oct4*) and *Sox2* are highly expressed in dividing rohu SSCs [21, 22], it would also be fascinating to carry out the association studies of *Nanog*, *Pou2* and *Sox2* in the regulatory mechanisms and pathways involved in proliferation and maintenance of teleost spermatogonial cells.

## Conclusions

In summary, the current study revealed the full-length cDNA sequence, genomic organization, and promoter characterization of *Nanog* gene of farmed rohu carp, *L. rohita*. By sequence comparison, phylogenetic analysis and genomic structure, the *LrNanog* was considered as the mammalian ortholog. The relatively conserved homeodomain present in *Nanog* of teleosts indicated that it might share some common biological functions with mammalian counterparts, particularly in stem cell maintenance. In support, we have provided evidence that *LrNanog* is transcriptionally active in proliferating SSCs and also up to the blastula stage of embryonic development. Its restricted abundances in adult gonads also confirmed its participation in stem cell proliferation and/or maintenance. The structural differences including overall conserved HOX domain and phylogenetic analyses highlighted its possible differential co-ordinating physiological functions between mammalian and teleostean germ cells development. Generation of DNA sequence information and its in silico analyses predicting several potential regulatory TFs in addition to the core promoter elements should provide a roadmap for undertaking future experiments linked to its regulated expression. Collectively, the results generated in this study validated the *LrNanog* as a potential biomarker for embryonic and SSCs and could be the first step towards elucidating mechanistic pathways in the stemness of SSC maintenance and proliferation in teleosts. Future studies could be carried out to validate functionally the regulatory mechanisms of *Nanog* gene in teleost species.

## Additional files

Additional file 1: Primers used for various PCR amplifications and mRNA expression analysis of Labeo rohita Nanog. (DOCX 14 kb)

Additional file 2: **Figure S1.** Schematic representation of different strategies opted to characterize the structural organization of rohu *Nanog* gene. Various fragments of *LrNanog* was obtained as illustrated by the closed and open boxes for the sense and antisense primers, respectively. The start and stop codons are represented as ATG and TGA, respectively. The genomic DNA is depicted with exons as rectangular boxes and the introns as solid lines. The exon-intron boundaries are determined by the alignment of cNDA sequence and genomic DNA sequence. RT-PCR, reverse transcription–polymerase chain reaction; UTRs, untranslated regions; RACE, rapid amplification of cDNA ends. (EPS 761 kb)

Additional file 3: **Figure S2.** Secondary structure of LrNanog protein predicted by SOPMA, confirming the absence of beta-sheets in *LrNanog* gene. (EPS 1350 kb)

Additional file 4: **Figure S3.** Ramachandran plot of LrNanog protein showing residues in most favored (white), additionally allowed (yellow), generously allowed (pale yellow), and disallowed region, generated by PROCHECK (SAVES, server). (EPS 2536 kb)

## Abbreviations

EGC: Embryonic germ cell
EM: Electrone microscopy
ESC: Embryonic stem cell
HOX: Homeobox
LIF: Leukemia inhibitory factor
ORF: Open reading frame
Plzf: Promyelocytic leukemia zinc finger
qPCR: Quantitative real time PCR
RACE: Rapid amplification of cDNA ends
SSC: Spermatogonial stem cell
TF: Transcription factor
UTR: Untranslated region
