# Supplementary material for: Molecular characterization and expression patterns of Nanog gene validating its involvement in the embryonic development and maintenance of spermatogonial stem cells of farmed carp, Labeo rohita
Source: J Anim Sci Biotechnol. 2018 Jun 11;9:45. doi: 10.1186/s40104-018-0260-2 (PMC5994655; doi:10.1186/s40104-018-0260-2)
Supplement: Supplementary file 1 — Primers used for various PCR amplifications and mRNA expression analysis of Labeo rohita Nanog. (DOCX 14 kb) [file 40104_2018_260_MOESM1_ESM.docx]

**Table S1** Primers used for various PCR amplifications and mRNA expression analysis of *Labeo rohita Nanog*.

| **Analysis methods** | **Primer Name** | **Primer Sequences** |
| --- | --- | --- |
| **CDS analysis** | LrNG.F | 5’- TCGCAGAACGGACGGCTCTTTC -3’ |
|  | LrNG.R | 5’- AGCGTGGCTGTGGGTAACTGGG -3’ |
| **RACE-PCR** | 5’-RACE-GSP1 | 5’- TCCCCAGGACGGAAGATTGAGGTTGGTC -3’ |
|  | 5’-RACE-GSP2 | 5’- TATGGGCCTCTGAATCACTGGGTGTA -3’ |
|  | 3’-RACE-GSP1 | 5’- TGCGTTTGTCTGACGACGCAATGG -3’ |
|  | 3’-RACE-GSP2 | 5’- AACGGCAGTGCCAGCACCTACG -3’ |
| **Genome Walking** | 5’-GW-GSP1 | 5’- TATGGGCCTCTGAATCACTGGGTGTA -3’ |
|  | 5’-GW-GSP2 | 5’- TGGCTTATCTTGTTTTCACGAGCTTGTGCA -3’ |
|  | 5’-GW-GSP3 | 5’- TCGACCTCCATAACCACAGCTGGCGCTGTT -3’ |
|  | 3’-GW-GSP1 | 5’- GCATACGGGCTCATGTACCCGCAAGTG -3’ |
|  | 3’-GW-GSP2 | 5’- AAGCTTTGCCTCCGCTTCCTGCATTGACCA -3’ |
| **RT-PCR** | *Nanog*.F | 5’- TGAATCTGGGGGTGGAAGCTTTGCCT -3’ |
|  | *Nanog*.R | 5’- TGTGCTGGGTACGCCATTGACGA -3’ |
|  | *β*-*actin*.F | 5’- ATCCTGACCGAGAGAGGCTACAG-3’ |
|  | *β*-*actin*.R | 5’- CCTTACGGATATCGACGTCAC-3’ |
|  | *Elf1α.*F | 5’-CTTCTCAGGCTGACTGTGC-3’ |
|  | *Elf1α.*R | 5’-CCGCTAGCATTACCCTCC-3’ |

bp; base pair
